# Fast-track transformation and genome editing in *Brachypodium distachyon*

**DOI:** 10.1186/s13007-023-01005-1

**Published:** 2023-03-29

**Authors:** Camille Soulhat, Houssein Wehbi, Yannick Fierlej, Patrick Berquin, Thomas Girin, Pierre Hilson, Oumaya Bouchabké-Coussa

**Affiliations:** Université Paris-Saclay, INRAE, AgroParisTech, Institut Jean-Pierre Bourgin (IJPB), 78000 Versailles, France

**Keywords:** *Brachypodium distachyon*, Genetic transformation, Genome editing, CRISPR/Cas9, Nitrate reductase (NR) mutants, Somatic embryogenesis

## Abstract

**Background:**

Even for easy-to-transform species or genotypes, the creation of transgenic or edited plant lines remains a significant bottleneck. Thus, any technical advance that accelerates the regeneration and transformation process is welcome. So far, methods to produce *Brachypodium distachyon* (*Bd*) transgenics span at least 14 weeks from the start of tissue culture to the recovery of regenerated plantlets.

**Results:**

We have previously shown that embryogenic somatic tissues grow in the scutellum of immature zygotic *Bd* embryos within 3 days of in vitro induction with exogenous auxin and that the development of secondary embryos can be initiated immediately thereafter. Here, we further demonstrate that such pluripotent reactive tissues can be genetically transformed with *Agrobacterium tumefaciens* right after the onset of somatic embryogenesis. In brief, immature zygotic embryos are induced for callogenesis for one week, co-cultured with *Agrobacterium* for three days, then incubated on callogenesis selective medium for three weeks, and finally transferred on selective regeneration medium for up to three weeks to obtain plantlets ready for rooting. This 7-to-8-week procedure requires only three subcultures. Its validation includes the molecular and phenotype characterization of *Bd* lines carrying transgenic cassettes and novel CRISPR/Cas9-generated mutations in two independent loci coding for nitrate reductase enzymes (*BdNR1* and *BdNR2*).

**Conclusions:**

With a short callogenesis stage and streamlined in vitro regeneration following co-cultivation with *Agrobacterium*, transgenic and edited T0 *Bd* plantlets can be produced in about 8 weeks, a gain of one to two months compared to previously published methods, with no reduction in transformation efficiency and at lower costs.

**Supplementary Information:**

The online version contains supplementary material available at 10.1186/s13007-023-01005-1.

## Background

Since the 1980s, the plant genetics toolbox has been vastly expanded in large part thanks to methods for the expression of synthetic genes inserted into plant chromosomes, mainly via direct DNA transfer into cell nuclei (e.g. through biolistics or protoplast transfection) or *Agrobacterium*-mediated transformation [29]. In recent years, we gained the ability to precisely engineer plant genomes with technologies based on transcription activator-like effector nucleases (TALENs) and clustered regularly interspersed short palindromic repeats (CRISPR)/Cas9 reagents [33]. However, the creation of transformants or edited lines for any plant species, or for any accession within a given species, is still hampered by our limited understanding of the factors that control the regeneration of in vitro cultured plant tissues into viable fertile adults [1]. Furthermore, even for genotypes that can be easily transformed, the creation of transgenic or edited higher plant lines producing seeds is always a fairly long process, lasting at least two months and sometimes years.

*Brachypodium distachyon* (hereafter *Bd*) has been chosen as the temperate C3 grass model species. It is closely related to wheat, barley and other cereal crops, but has a small genome (271 Mbp; [19] and a small stature. Abundant genetic resources are publicly available for *Bd* and it can be transformed via *Agrobacterium tumefaciens* [12, 28].

Several articles describe protocols for *Bd* genetic transformation (e.g. [2, 6, 8, 9, 31] and more recently gene editing [18, 26]. In most cases, the resulting lines are generated via *Agrobacterium*-mediated transformation of embryogenic tissues forming in the scutellum of immature zygotic embryos (izEmb). Overall, the time elapsed between the preparation of the initial explants and the transfer of *Bd* plantlets, transgenic or edited, onto a rooting medium is between 13 and 16 weeks.

We have recently shown that embryogenic meristematic tissues develop in as little as three days within *Bd* izEmb scutellum explants exposed to the synthetic auxin 2,4-dichlorophenoxyacetic acid (2,4-D) and that the regeneration of *Bd* plantlets can be initiated immediately thereafter by further treating the three-day explants with an exogenous cytokinin [35]. Reasoning that such a short sequential procedure could help accelerate the creation of transgenic and edited *Bd* lines, we adapted our fast regeneration protocol to include *Agrobacterium* co-cultivation and in vitro selection of transformed tissues. In our hands, *Bd* plantlets carrying a transgene or CRISPR/Cas9-induced mutations can be transferred onto a rooting medium within seven to eight weeks, thus shortening published methods by five to seven weeks. Our results indicate that the rapid regeneration/transformation method detailed herein is as robust and efficient as those we previously implemented for the creation of either transgenic or edited *Bd* individuals.

## Results and discussion

### Streamlined regeneration of transgenic *Bd* plants

**Fig. 1:**
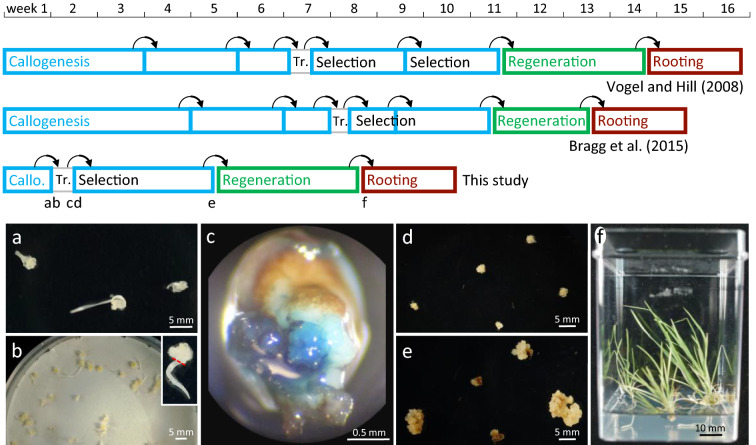
Overview of the rapid transformation protocol. Two reference *Bd* transformation protocols [6, 32] are represented on top for comparison to the method described in this study, developed for the accession Bd21-3. Boxes in blue, in vitro culture steps on Callus Induction Medium (CIM); in green, on Shoot Induction Medium (SIM); in brown, on rooting medium. Arrows, steps involving explant transfer between plates. Tr., transformation via coculture of plant tissues with *Agrobacterium tumefaciens* strains. **a** Immature zygotic embryo (izEmb) explants after one week on CIM. **b** Explants co-cultivated for three days on paper filter with *Agrobacteirum*. Inset shows where elongated coleoptiles are chopped off (red dotted line) prior transfer onto selective CIM. **c** GUS-stained izEmb after agroinfiltration showing that embryogenic proliferating tissues protruding from the scutellum transiently express the reporter gene. **d** Transformed izEmb explants at the start of the 3-week hygromycin selection. **e** Resulting calli at the end of the selection. **f** Regenerated plantlets at the end of in vitro rooting

Based on previous results [35], we aimed to shorten our *Brachypodium distachyon* transformation protocol. To highlight the original features of the method presented here, we compare its successive stages to two reference protocols published by Vogel and coworkers [6, 32] whose timeframe is very similar to that of other reports [2, 8, 9] (Fig. 1).

First, we considered bypassing altogether the initial 6-to-7-week callus multiplication. By trials and errors, we determined that 2,4-D-induced embryogenic cells can be efficiently transformed via co-cultivation with *Agrobacterium* starting only seven days after the onset of tissue culture (Fig. 1a) on Callus Inducing Medium (CIM). Earlier co-cultivation, after three days of izEmb incubation, prevented the recovery of any transgenics*.*

To monitor agrotransformation at successive steps of the procedure, plant tissues were tested for β-glucuronidase activity in overnight X-Gluc assays, following 3-day co-cultivation with *Agrobacterium* (Fig. 1b) carrying a T-DNA with a *GUS* transgene (vector pIPKb2GUS). In izEmb explants tested immediately after co-cultivation, young meristematic tissues showed deep blue staining confirming the efficient transfer of T-DNA into pluripotent plant cells (Fig. 1c).

Once transformed, the proliferative tissues evolved as expected on selective CIM, with hygromycin-resistant callus bulges growing from actively dividing sectors. Because izEmb explants are still quite small after a 7-day 2,4-D induction, the selection of transgenic proliferative tissues on CIM containing hygromycin (40 mg.L^−1^) could be limited to three weeks, with no transfer on fresh plates (Fig. 1d, e), compared to previous methods including a subculture after one or two weeks for a total of three to four weeks of selective CIM incubation (see timelines in Fig. 1).

Thereafter, embryogenic calli switched onto Shoot Inducing Medium (SIM) containing cytokinin rapidly yielded transgenic regenerants that rooted normally and further developed as fertile adult plants (Fig. 1f)*.* The rate of non-transformed regenerated plantlets (escapes) we observed was very low (2/40) amongst the plantlets recovered after co-cultivation with a *GUS* T-DNA *Agrobacterium* strain.

### Genetic and molecular characterization of *Bd* transgenics

To confirm stable transformation and assess segregation patterns, we analyzed GUS activity in the T1 progeny of selfed T0 plants. As expected, various GUS segregation patterns were observed. Among 13 randomly selected plants, eight present segregation ratios suggesting that they carry a single T-DNA insertion, two that they carry multiple insertions, and one other has a ratio compatible with a 2:1 pattern possibly explained by skewed gamete transmission (Table 1; χ^2^ test, P < 0.05). For the two last plants analyzed, ratios may be coherent with a single T-DNA insertion or other configurations that could be distinguished with larger T1 progeny samples. The presence of the *HptII* gene coding hygromycin resistance was confirmed in the recovered transformants (Additional file 1: Fig. S1).

**Table 1:**
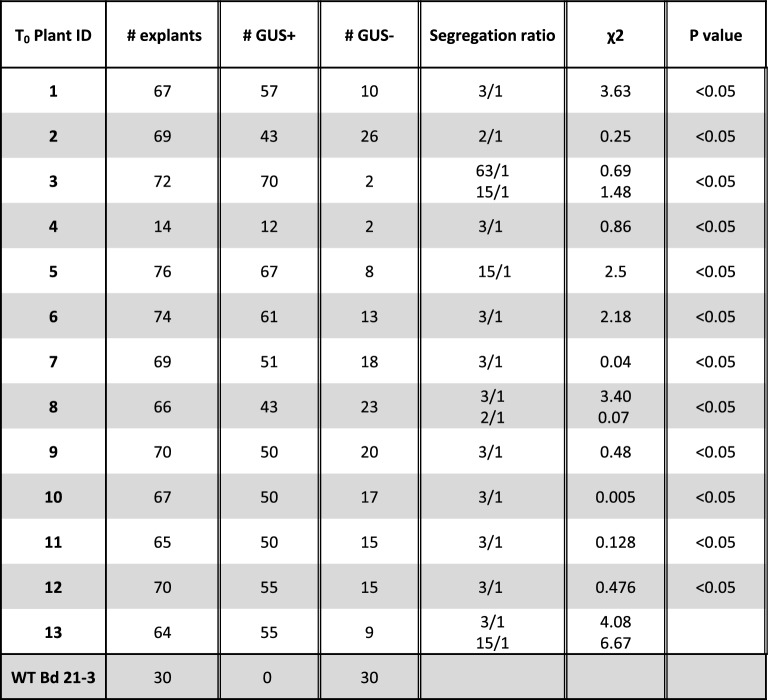
Segregation results in the F1 progeny of recovered T0 plants transformed with pIPKb2GUS

Blue stained embryos were recorded as GUS +  and unstained embryos as GUS−

In summary, our rapid protocol involves three subculture steps (instead of six in previous reports) and lasts 10 weeks from the initiation of tissue culture to the recovery of rooting transformed plantlets. The resulting transformation efficiency (number of regenerated transgenic plantlets over the number of sampled izEmb explants) is ~ 16% (Bd21-3 accession), similar to the efficiency we routinely achieve with the Vogel and Hill [32] protocol.

### Rapid production of CRISPR/Cas9-induced mutants

Site-directed mutagenesis has become a very powerful tool for functional analyses: mutations can be created at a precise locus and mutations at multiple loci can be combined at once, thus greatly facilitating complex genetic studies. We chose the *Nitrate Reductase* (*NR*) genes as proof-of-concept targets to demonstrate that the transformation method we developed is useful to rapidly and efficiently produce *Bd* mutants via the CRISPR/Cas9 technology. Similarly, the *NR1* gene from *Arabidopsis thaliana* has very recently been used as proof-of-concept target for transgene-free genome editing by grafting [38]. NR proteins are key enzymes of nitrate assimilation in higher plants [3] and are also indirectly involved in cell signaling [23, 39]. While never described in *Bd* to our knowledge, *nr* loss-of-function mutants have been extensively characterized in several plant species, including *Arabidopsis thaliana* [5, 36, 37] and monocotyledonous crops [11, 13, 16, 34], thereby providing background knowledge to interpret potential mutant phenotypes.

**Fig. 2:**
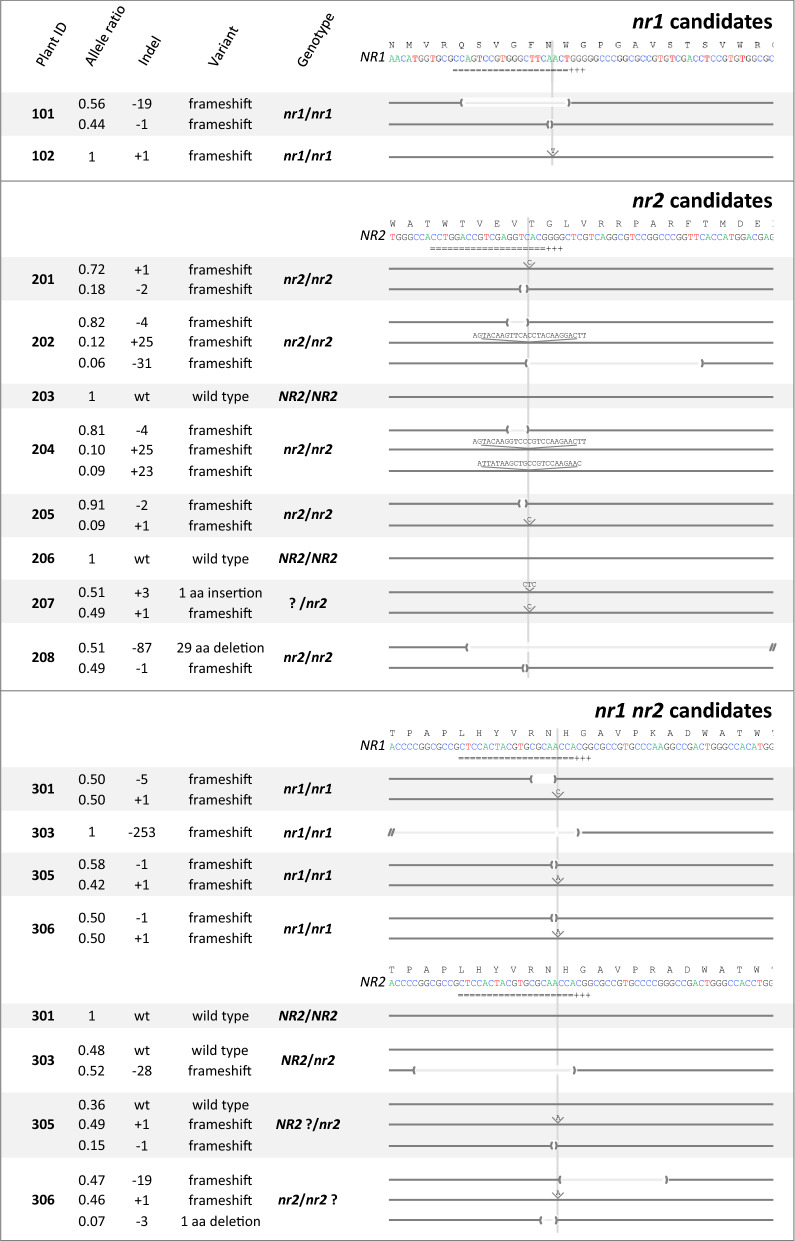
Graphic representation of CRISPR/Cas9-generated *BdNR* mutant alleles. The gRNA target site is represented under each wild-type DNA sequence, “ + ” signs underlining the Cas9 protospacer adjacent motif (PAM). Grey vertical lines indicate the position of predicted Cas9 cleavage sites. Deleted nucleotides are highlighted between parentheses, inserted ones are listed and positioned on top of the corresponding allele. All sequences were deconvoluted with the DECODR algorithm [4] based on Sanger chromatographs generated from both DNA strands, except for plant #101 for which only ( +) strand chromatographs yielded predicted alignment with the wild-type *NR1* sequence and plant #303 for which the large *NR1* deletion (− 253 nucleotides) was positioned manually

Through Blast similarity searches with the sequences of AtNIA1 and AtNIA2, known *Arabidopsis thaliana* NR proteins [37], we identified two likely Nitrate Reductase coding orthologs in the genome of *Brachypodium distachyon*: Bradi3g57680 and Bradi3g37940, named here *BdNR1* and *BdNR2*, respectively. Both putative *BdNR* genes are expressed in seeds, roots, leaves and internodes of adult *Bd* plants, *BdNR2* at a higher level in the three latter tissues (http://bar.utoronto.ca/efp_brachypodium/). As a first step to investigate their role and potential redundancy, we created single mutant lines for each of them, as well as double mutant lines. For this purpose, we designed three Cas9 guide RNAs (gRNAs): two were designed in non-conserved regions specific to either gene, the third was designed to introduce mutations in a strictly conserved region shared by both (Fig. 2).

Each gRNA coding sequences was cloned in a binary vector containing the Cas9 cassette (pHUbi-Cas9-9.7) and transformed separately via *Agrobacterium* according to the method described above. Three independent transformations (235 transformed izEmb) were required to recover two *nr1* T0 regenerant candidates, another (112 izEmb) yielded eight *nr2* putative mutants and a final experiment produced six *nr1 nr2* candidates (162 izEmb), respectively. Selected plantlets were rooted and transferred to the greenhouse. Whenever possible, green leaves were sampled from the regenerants for molecular analysis and NR activity assays. The presence of *HPTII* and *Cas9* sequences was confirmed in all analyzed plants (Additional file 1: Fig. S2).

To assess the presence of Cas9-induced mutations, the *BdNR1* and *BdNR2* targeted regions were PCR amplified and Sanger sequenced. The resulting chromatographs were compiled and compared with the DECODR software [4] with the aim (i) to detect inserted or deleted nucleotides at or near the predicted Cas9 cleavage sites and (ii) to measure the relative fraction of the different alleles in each separate plant. Overall, in 18 regenerated T0 plants, we identified 10 distinct mutant alleles in *BdNR1* and 13 in *BdNR2*, all consisting in indels of varying sizes, close to the Cas9 cleavage site, and entirely contained within the first exon of either gene (Fig. 2). All indels resulted in a frameshift, except *BdNR2* alleles that included a codon insertion (in plant #207) or the deletion of a single (#306) or 29 codons (#208) (Fig. 2). In 13 plants, the allele ratio suggests simple biallelism. In the others, the *BdNR2* allele ratio is markedly different from 1:1 (#201, #205) or three *NR2* alleles were detected (#202, #204, #305, #306) which could be explained by PCR biases or chimerism due to CRISPR/Cas9 activity in the characterized leaves. Based on the deconvoluted allele sequences and the nature of the resulting mutant variants, the genotyped plants comprise two homozygous *nr1*/*nr1 NR2*/*NR2* individuals (#101, #102, #301), five *NR1*/*NR1 nr2*/*nr2* (#201, #202, #204, #205, #208), one *nr1*/*nr1 NR2*/*nr2* (#303) and possibly one homozygous *nr1*/*nr1 nr2*/*nr2* double mutant (#306) (Fig. 2).

**Fig. 3:**
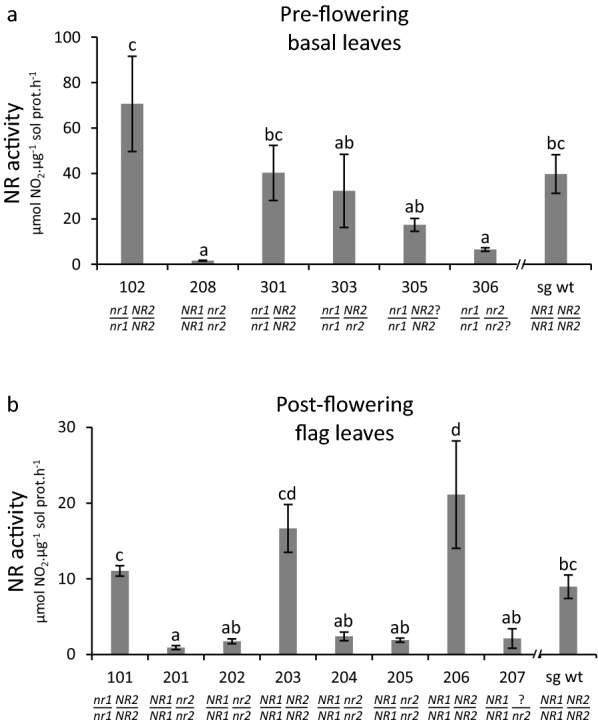
Nitrate reductase activity in CRISPR/Cas9-generated mutants. NR activity was measured in a pool of three leaves sampled from each analyzed plant at the pre-flowering (**a**) or post-flowering (**b**) stage. Plant numbers and genotypes are as in Fig. 2. Seed-grown wild-type (sg wt) plants are controls at a similar growth stage, but with a different development history than the regenerants. Average ± SD are shown (n = 3). Letters represent significant differences in a 95% family-wide confidence level comparison

Recovered T0 *nr* mutant candidates were analyzed for Nitrate Reductase (NR) activity in two batches: one focused on pre-flowering basal leaves (Fig. 3a), the other on post-flowering flag leaves (Fig. 3b). First note that *BdNR1 BdNR2* wild-type regenerants show higher NR activity than seed-germinated wild-type plants uniquely grown in soil (compare “sg wt” to #203 and #206 in Fig. 3b) possibly because they have a markedly different development history. NR activity is drastically reduced in *nr2* homozygous leaves (#201, #202, #204, #205, #208), while *nr2* hemizygous leaves show intermediate activity (#303). The intermediate NR activity also measured in leaves of plants #207 and #305, together with the drastically reduced NR activity in #306, further suggests that the three in-frame *BdNR2* indel alleles may result in partial or total loss-of-function. In contrast, in single *nr1* homozygous tissues (pre-flowering basal leaves, #102, #301; post-flowering flag leaves, #101), NR activity is not consistently different from that in seed-germinated (sg wt) or regenerated wild-type leaves (#203, #206). Hence, whether *BdNR1* is active or not in leaf tissues remains an open question.

Our initial biochemical results indicate that, despite their sequence similarity, *BdNR1* and *BdNR2* do not seem to contribute equally to leaf NR activity. Two *NR* genes have also been described in most characterized angiosperms [24]. When further characterized in Arabidopsis [37] and rice [15], such paired *NR* genes appear to code for distinct isoforms that are only partially redundant, in agreement with our observations.

The *Bd nr* mutant plants we recovered did not show obvious growth or developmental defects. They were grown on culture media or watered with a nutrient solution containing ammonium salts, in conditions that prevent the development of nitrogen deficiency phenotypes. Thus, further analysis is required to establish the detailed role of each *BdNR* gene in nitrate metabolism and signaling.

### Conclusion

In comparison to previous reports, the protocol presented herein cuts one to two months to the creation of *Bd* transgenics [6, 32] or CRISPR/Cas9-induced mutants [18, 22, 27], with no observable reduction in transformation efficiency. In routine procedures, we typically use ca. 100 immature primary embryos in a single *Agrobacterium* co-cultivation experiment for each construct of interest (five initial CIM plates, each with 20 izEmb), whether for straightforward transgenesis or simple CRISPR/Cas9 gene editing. In our laboratory, a trained operator collects 100 precisely-staged *Bd* izEmb within three to six hours. Thus, hands-on time dedicated to in vitro culture manipulations is equivalent for this fast-track protocol and previously described methods, since the latter require the dissection of fewer immature zygotic embryos but include three additional CIM subcultures. The rapid transformation protocol also cuts costs because it entails smaller medium volume, fewer culture plates and shorter in vitro culture room occupancy.

The transformation of immature zygotic embryos at—or shortly after—the onset of in vitro culture has already been implemented in different plant species, for example in bread wheat for biolistic or *Agrobacterium*-mediated transformation [20, 30]. This report further shows that established protocols taking advantage of induced somatic embryogenesis may be revisited to improve transformation or gene editing in a wide range of genotypes and plant species.

## Methods

### Plant materials and embryogenic callus induction

*Brachypodium distachyon* mother plants (accession Bd21-3) were maintained in a growth chamber with a cycle of 4 h darkness followed by 20 h of light (OSRAM Lumilux L36W865 cool day light; 320 µmol.m^−2^.s^−1^), at 60% hygrometry and 24 °C. Spikes were sampled ~ 7 weeks after germination for extraction of immature embryos at a precise developmental stage identified as follows: spikes are still green, the anthers are visible outside the inflorescences and seeds are tender but well filled. Spikes were sterilized for 30 min in a 800 mL solution containing 1.5 g active chlorine stirred with a magnetic bar, and finally rinsed twice in sterile water. Only transparent immature embryos of ~ 400–600 µm were collected and transferred on gelled Callus Induction Medium (CIM) in 90 mm round Petri dishes. CIM plates were incubated for one week in a growth chamber at 70% hygrometry and 28 °C, in the dark.

### *Agrobacterium* strains and constructs

The vector pIPKb2 GUS Intron was obtained by Gateway recombination (Life technology) of pEN-L1-SI-L2 [21] into pIPKb2 destination vector containing the maize ubiquitin promoter [17].

The CRISPR/Cas9 constructs were previously described [25]. The T-DNA in destination vector pHUbi-Cas9-9.7 contains expression cassettes for Cas9 and gRNA. The gRNAs were designed with the CRISPOR software [10]: CTCCACTACGTGCGCAACCA targets both *bdNR1* and *bdNR2*, CCAGTCCGTGGGCTTCAACT *bdNR1* and CCTGGACCGTCGAGGTCACG *bdNR2.* All sites lay in their first exon. Double-stranded oligonucleotides corresponding to each gRNA, flanked with *BsaI* restriction sites, were cloned into the pOssgRNA vector [25], then introduced in pHUbi-Cas9-9.7 by LR Gateway recombination. All vectors were validated by sequence analysis and electroporated into the *AGL1 Agrobacterium tumefaciens* strain.

### Tissue transformation, selection and growth conditions

For each transformation, a stationary *Agrobacterium* suspension was prepared in the co-culture medium at OD_600_ = 0.3. Callogenic *Bd* immature embryos, previously incubated for one week on CIM, were collected and immersed in the bacterial suspension for 5 min, in a 50-mL Falcon tube. The liquid suspension was discarded by pouring the mix over a filter. The next few steps were designed to remove excess bacterial suspension, enabling efficient plant cell transformation while avoiding bacterial overgrowth during tissue culture. The retained imbibed callogenic embryos were set to dry in the laminar flow hood on a sterile Whatman filter paper for 20 min. The explants were then transferred in Petri dishes containing a round Whatman sterile filter paper humidified with 500 µL of co-culture medium. The Petri dishes were sealed with two rounds of Parafilm and incubated in the dark, at 24 °C, for a 3-day co-cultivation. Embryos were then transferred to selective CIM. As a fraction of the cultured immature zygotic embryos germinate during the first week on CIM (Fig. 1a, b), their extended coleoptile was chopped off with a scalpel prior transfer on selection medium (inset in Fig. 1b). Selective CIM plates were closed with Parafilm and incubated at 28 °C, with 70% hygrometry in the dark for 3 weeks.

### Regeneration, rooting and transfer to soil

At the end of the 3-week selection period, only potentially transformed embryos which evolved into callus were transferred to regeneration Shoot Induction Medium (SIM). A few calli (6–8) were plated on each Petri dish with ample room for them to grow. Dishes sealed with two rounds of Parafilm were incubated with a 16 h light photoperiod (60 mol.m^−2^.s^−1^ intensity) at 28 °C and 70% hygrometry. Regenerating plantlets grown on SIM until approximately 2 cm in length were then transferred onto rooting medium Magenta boxes, and eventually into soil (10 cm pot), first under a plastic cover until they resumed growth. The detailed composition of all in vitro culture media is provided in Additional file 2: Table S1.

### Molecular and histochemical characterization of transgenic and mutant *Bd* plants

To test for GUS activity, 14 random independent T0 transgenics were sown and T1 plantlets were screened together with controls. T1 embryos or leaf fragments were sampled, vacuum-infiltrated for 30 min in GUS X-Gluc substrate buffer, and incubated at 37 °C overnight. Explants were scored for blue staining after rinsing twice in 96% ethanol.

For DNA extraction, leaves were sampled from each regenerant and ground in 2 ml Eppendorf tubes with a FastPrep-24 homogenizer for 5 min. DNA was isolated using the CTAB buffer. Transgene presence was checked by PCR amplification of *Cas9* and *HptII* genes. The presence of mutations in *BdNR1* and *BdNR2* was detected by PCR amplification of the target sites followed by Sanger sequencing. Specific primer sequences are listed in Additional file 3: Table S2. Sequence analysis was performed through deconvolution of ABI chromatograph data and alignment to the reference sequences with DECODR analysis tool (https://decodr.org/; [4]).

### Measurement of nitrate reductase activity

According to a protocol adapted from Ferrario-Méry et al. [14], three leaf samples were sampled from each transformant, frozen-ground and stored at − 80 °C until extraction of soluble proteins. The extraction buffer consisted of 50 mM MOPS-KOH, pH 7.6, 1 µM Na_2_MoO_4_, 10 µM FAD, 4 µM leupeptin, 0.2 g/g fresh weight PVP, 2 mM β-mercaptoethanol, and 5 mM EDTA. Crude homogenates were then centrifuged for 5 min at 12,000 g and 4 °C. Supernatant NR activity was assayed immediately in a reaction mix containing 50 mM MOPS-KOH buffer, pH 7.6, 10 mM KNO_3_, 0.155 mM NADH and 5 mM EDTA. The enzymatic reaction was stopped after 15 min of incubation at 30 °C with the addition of an equal volume of sulfanilamide (1%, w/v in 3 N HCl) followed by an equal volume of sulfanilamide of n*-*napthyl ethylenediamine dihydrochloride (0.02%, w/v), and *A*_540_ was measured. NR activity in each leaf extract was normalized relative to soluble protein content measured with the Bio-Rad protein assay based on the Bradford dye-binding method [7]. Three technical repeats were performed for NR activity and protein content for each sample. Technical repeat measurements were averaged before normalizing NR activity by protein content. Statistical analysis was performed on the three normalized values per transformant.

### Statistical analysis

A Chi-squared (χ^2^) test was applied to determine whether the difference between the observed number of GUS + plantlets in the T1 population and the expected segregation pattern was statistically significant. Results were interpreted with one degree of freedom and a P value < 0.05. Comparison of nitrate reductase activity between *nr* mutant candidate lines and wild type was performed with a one-way ANOVA test and a 95% family-wise confidence level comparison, using the Rcmdr package of the R software (http://cran.r-project.org/web/packages/Rcmdr/).

## Supplementary Information

**Additional file 1: Fig. S1.** Detection of transgene sequences in *Bd GUS* transformants. Agarose gel (1%) for *HptII* PCR amplicon analysis. Lanes 1 and 17: 1kb Plus DNA Ladder (Invitrogen); lanes 2 to 13: bulked T1 progeny of plants listed in Table 1; lanes 14 and 15: negative controls; lane 16: positive control. **Fig. S2.** Detection of transgene sequences in T0 *Bd NR* mutant candidate regenerants. **a** Agarose gel (1%) for *HptII* PCR amplicon analysis. Lanes 1 and 19: 1kb Plus DNA Ladder (Invitrogen); lanes 2 to 15: CRISPR/Cas9-induced *NR* mutant candidates represented in Fig. 2; lanes 16 and 17: negative controls; lane 18: positive control. **b** Agarose gel (1%) for *Cas9* PCR amplificon analysis. Same configuration as above.

**Additional file 2: Table S1.** Media composition per liter.

**Additional file 3: Table S2.** Gene-specific primers for PCR amplification and sequencing.

## Data Availability

All data generated or analyzed during this study are included in this published article and its supplementary information files.

## Abbreviations

*Bd*: *Brachypodium distachyon*
CIM: Callus Induction Media
CTAB: Cetyltrimethylammonium bromide
EDTA: Ethylenediaminetetraacetic acid
IBA: Indole-3 butyric acid
izEmb: Immature zygotic embryo
NR: Nitrate Reductase
SIM: Shooting Induction Media
2,4-D: 2,4-Dichlorophenoxyacetic acid
