# Supplementary material for: Fast-track transformation and genome editing in Brachypodium distachyon
Source: Plant Methods. 2023 Mar 29;19:31. doi: 10.1186/s13007-023-01005-1 (PMC10053978; doi:10.1186/s13007-023-01005-1)
Supplement: Supplementary file 1 — Additional file 1: Fig. S1. Detection of transgene sequences in Bd GUS transformants. Agarose gel (1%) for HptII PCR amplicon analysis. Lanes 1 and 17: 1kb Plus DNA Ladder (Invitrogen); lanes 2 to 13: bulked T1 progeny of plants listed in Table 1; lanes 14 and 15: negative controls; lane 16: positive control. Fig. S2. Detection of transgene sequences in T0 Bd NR mutant candidate regenerants. a Agarose gel (1%) for HptII PCR amplicon analysis. Lanes 1 and 19: 1kb Plus DNA Ladder (Invitrogen); lanes 2 to 15: CRISPR/Cas9-induced NR mutant candidates represented in Fig. 2; lanes 16 and 17: negative controls; lane 18: positive control. b Agarose gel (1%) for Cas9 PCR amplificon analysis. Same configuration as above. [file 13007_2023_1005_MOESM1_ESM.pptx]

## Slide 1
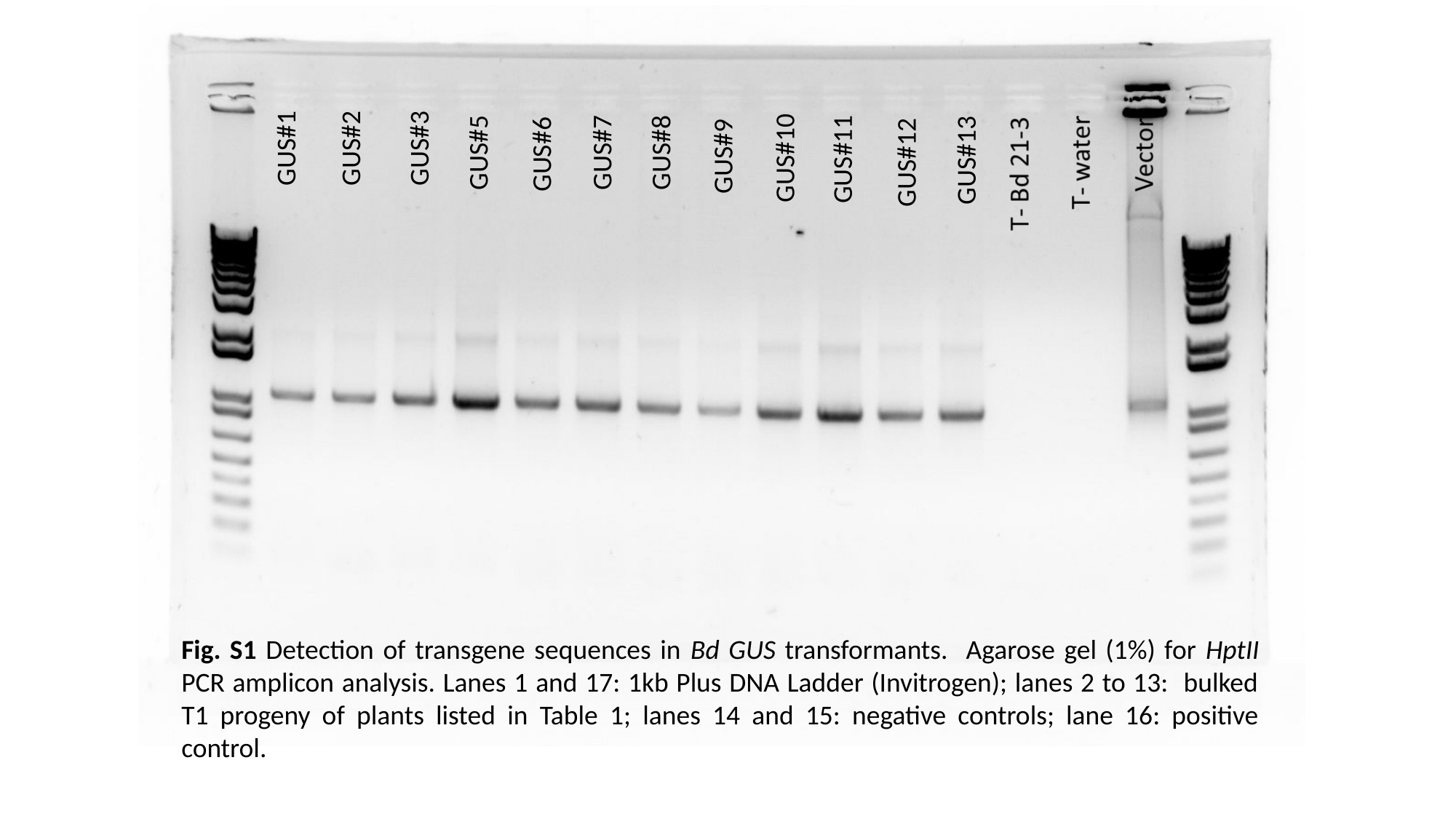

GUS#2
GUS#11
GUS#7
GUS#13
GUS#8
GUS#10
GUS#1
GUS#3
GUS#6
GUS#9
GUS#5
GUS#12
Fig. S1 Detection of transgene sequences in Bd GUS transformants. Agarose gel (1%) for HptII PCR amplicon analysis. Lanes 1 and 17: 1kb Plus DNA Ladder (Invitrogen); lanes 2 to 13: bulked T1 progeny of plants listed in Table 1; lanes 14 and 15: negative controls; lane 16: positive control.

## Slide 2
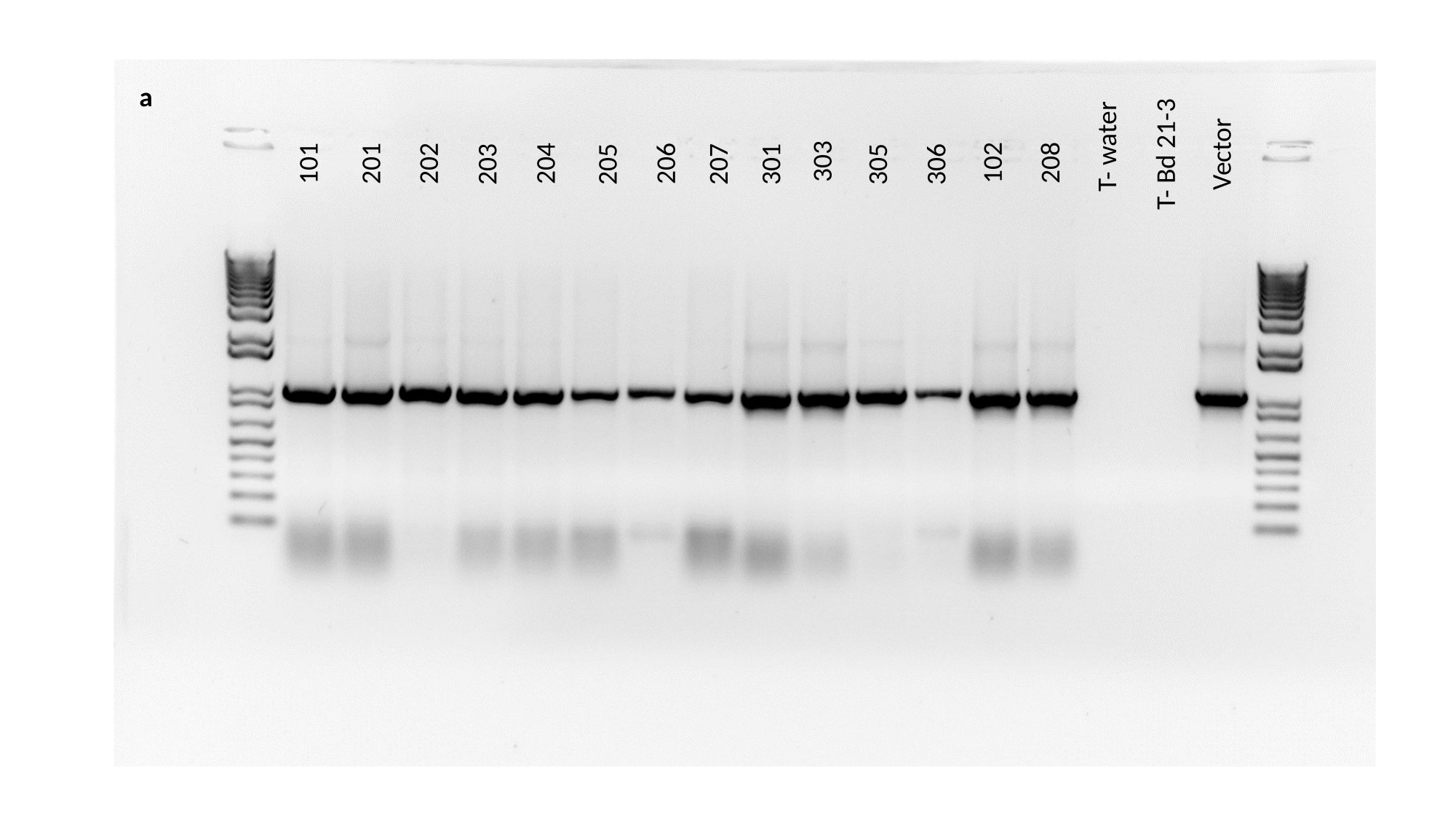

201
Vector
206
204
101
202
203
205
207
306
303
208
T- water
102
301
305
T- Bd 21-3
a

## Slide 3
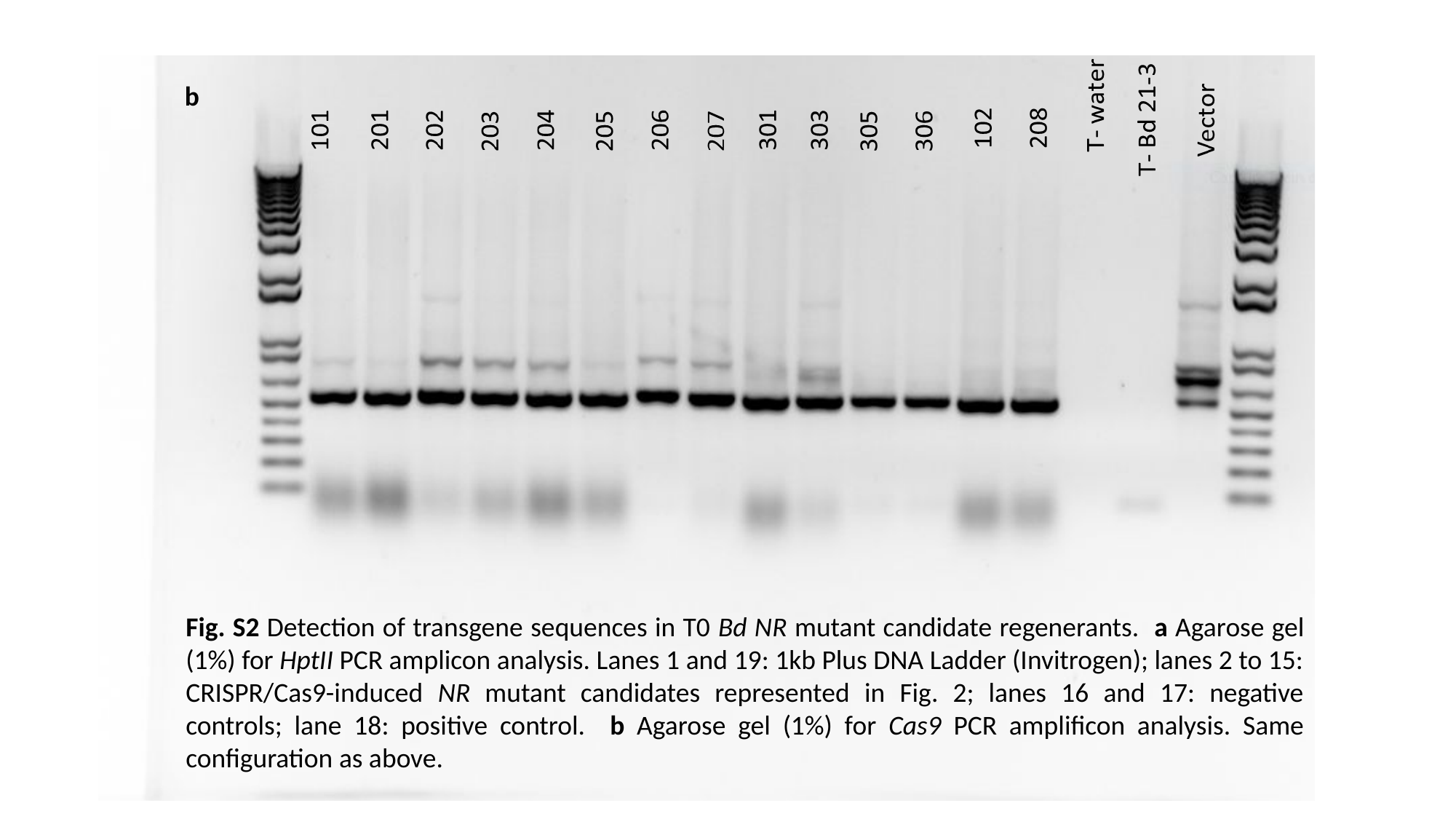

b
Fig. S2 Detection of transgene sequences in T0 Bd NR mutant candidate regenerants. a Agarose gel (1%) for HptII PCR amplicon analysis. Lanes 1 and 19: 1kb Plus DNA Ladder (Invitrogen); lanes 2 to 15: CRISPR/Cas9-induced NR mutant candidates represented in Fig. 2; lanes 16 and 17: negative controls; lane 18: positive control. b Agarose gel (1%) for Cas9 PCR amplificon analysis. Same configuration as above.
