# Supplementary material for: Fast-track transformation and genome editing in Brachypodium distachyon
Source: Plant Methods. 2023 Mar 29;19:31. doi: 10.1186/s13007-023-01005-1 (PMC10053978; doi:10.1186/s13007-023-01005-1)
Supplement: Supplementary file 2 — Additional file 2: Table S1. Media composition per liter. [file 13007_2023_1005_MOESM2_ESM.docx]

**Table S1:** Media composition per liter.

|  | **Basal medium** | **CIM** | **Cocu** | **Selec** | **SIM** | **Rooting** |
| --- | --- | --- | --- | --- | --- | --- |
| **MS (Duchefa, M0222)** | 4.4 g | 4.4 g | 4.4 g | 4.4 g | 4.4 g | 4.4 g |
| **Sucrose (Duchefa, S0809)** | 30 g | 30 g | 30 g | 30 g | / | 10 g |
| **Maltose (Duchefa, M0811)** | / | / | / | / | 30 g | / |
| **CuSO_4_.5H_2_O (Sigma, C3036, 0.6 mg/mL)** | 1 ml | 1 ml | 1 ml | 1 ml | / | / |
| **Phytagel (Sigma P8169)** | 2 g | 2 g | / | 2 g | 2 g | 2 g |
| **2,4 D (Sigma, D7299, 2.5 mg/mL)** | / | 1ml | / | 1 ml | / | / |
| **Kinetin (Sigma, K0905, 0.4mg/ml)** | / | / | / | / | 1 ml | / |
| **IBA (Sigma, I5386, 5 mg/ml)** | / | / | / | / | / | 1 ml |
| **Acetosyringone (Sigma, D134406, 40 mg/mL)** | / | / | 1 ml | / | / | / |
| **Hygromycin (Sigma, H3274, 40 mg/ml)** | / | / | / | 1 ml | 1 ml |  |
| **Carbenicillin (Duchefa, C0109, 250mg/ml)** | / | / | / | 1 ml | 1 ml | / |
| **Cefotaxim (Duchefa, C0111, 200 mg/ml)** | / | / | / | 400 μl | 400 μl | / |
|  |  |  |  |  |  |  |

The pH was adjusted at 5.7 before steam sterilization. Phytohormones and antibiotics were filtered-sterilized and added after sterilization.
