# Supplementary material for: Fast-track transformation and genome editing in Brachypodium distachyon
Source: Plant Methods. 2023 Mar 29;19:31. doi: 10.1186/s13007-023-01005-1 (PMC10053978; doi:10.1186/s13007-023-01005-1)
Supplement: Supplementary file 3 — Additional file 3: Table S2. Gene-specific primers for PCR amplification and sequencing. [file 13007_2023_1005_MOESM3_ESM.docx]

**Table S2.** Gene-specific primers for PCR amplification and sequencing.

| **Gene** | **Primer sequences (5' > 3')** | **Product length (bp)** | **Tm (°C)** |
| --- | --- | --- | --- |
| *CAS9* | Fwd-CCTTGACGGACTTGAGCTTC | 561 | 58 |
|  | Rev-TGCGCGAGATCAACAACTAC |  | 58 |
| *HptII* | Fwd-ATGAAAAAGCCTGAACTCACCGCGAC | 1214 | 58 |
|  | Rev-CTATTTCTTTGCCCTCGGACGAGTGC |  | 58 |
| *BdNR1* | Fwd-ACTCCGACGACGAAGACAAC | 553 | 58 |
|  | Rev-GCACCTTCAAAGCACACGTT |  | 58 |
|  | Fwd2-ATTACAACCGTCAGGCGTCC | 1339 | 58 |
|  | Rev2-GCCCAAAACAAGTCCCGAGA |  | 58 |
| *BdNR2* | Fwd-GCTTCTCCCCGCTCATATC | 711 | 58 |
|  | Rev-CATGTAGGCGAGCATGATGT |  | 58 |
